# Low BMI and weight loss aggravate COPD mortality in men, findings from a large prospective cohort: the JACC study

**DOI:** 10.1038/s41598-020-79860-4

**Published:** 2021-01-15

**Authors:** Hiroo Wada, Ai Ikeda, Koutatsu Maruyama, Kazumasa Yamagishi, Peter J. Barnes, Takeshi Tanigawa, Akiko Tamakoshi, Hiroyasu Iso

**Affiliations:** 1 Department of Public Health, Juntendo University Graduate School of Medicine, Bunkyo, Tokyo Japan; 2 Department of Bioscience, GraduateSchool of Agriculture, Ehime University, Matsuyama, Ehime Japan; 3 Department of Public Health Medicine, Faculty of Medicine, and Health Services Research and Development Center, University of Tsukuba, Tsukuba, Ibaraki Japan; 4 National Heart and Lung Institute, Imperial College London, London, UK; 5 Department of Public Health, Hokkaido University, Sapporo, Hokkaido Japan; 6 Department of Social and Environmental Medicine, Graduate School of Medicine, Osaka University, Suita, 2-2 Yamada-oka, Suita, Osaka 565-0871 Japan

**Keywords:** Environmental social sciences, Risk factors

## Abstract

To clarify how low BMI and weight loss were associated with risk of chronic obstructive pulmonary disease (COPD) mortality, in a large prospective cohort of the general population across Japan, the Japan Collaborative Cohort Study, conducted between 1988 and 2009. A total of 45,837 male residents were observed for a median period of 19.1 years. Self-administered questionnaires, collecting information on BMI, weight loss since the age of 20, lifestyles, history of diseases, as well as records of COPD mortality, were analysed at 2019. During follow-up, 268 participants died from COPD. The multivariate-adjusted hazard ratio (95% confidence interval) of COPD mortality associated with a 1-SD increment of body mass index (BMI) was 0.48 (0.41–0.57), while for weight change from age of 20 (+ 2.0 kg) it was 0.63 (0.59–0.68). These associations were persistently observed after stratifications with smoking status, excluding those having airway symptoms in the baseline survey, and excluding early COPD deaths within 5, 10 and 15 years. Our study suggests that BMI and weight change since the age of 20 could be markers for COPD prognosis, indicated by risk of COPD mortality.

## Introduction

Increasing mortality caused by chronic obstructive pulmonary disease (COPD) has become an emerging medical and public health issue in ageing societies such as Japan and many Western countries. Follow-up studies of COPD patients^1–7^ and meta-analyses of case–control studies^8,9^ have shown an association between COPD patients having low BMI and lower survival rates. However, the generalizability of these studies is limited, because the data have been analysed between underweight COPD patients and those who were not. Many previous studies were conducted in Western countries^1–6^, whilst only a few reports were from Japan^7^ where the mean and distribution of body mass index (BMI) are much lower. In 2010, only 3.3% of the Japanese population had a BMI of 30 kg/m^2^ or higher, whereas individuals with a BMI over 30 represented 32.3% of the United States and 24.2% of the United Kingdom according to the WHO statistics^10^.

Only a single epidemiological study from China referred to the association between having low BMI and mortality from COPD^11^, although COPD accounted for as high as 87% of the respiratory mortality in China^11^, different from those in Korea, i.e. asthma and tuberculosis accounted for 24.7% and 19.3% of respiratory mortality respectively^12^. In contrast, pneumonia, COPD and asthma accounted for 64.0%, 8.8% and 1.2%, respectively in Japanese men in 2009^13^.

In the present study, we investigated how low BMI and weight loss are associated with COPD mortality in a large prospective cohort of middle-aged Japanese men.

## Results

Table 1 shows participants’ age-adjusted baseline characteristics according to BMI. Compared to the control group whose BMI was 20.0 to < 22.0 kg/m^2^, those with BMI < 18.5 kg/m^2^ were more likely to be older and current smokers, to walk 1 h or more per week, and to have histories of diabetes. Those with BMI ≥ 22.0 kg/m^2^ were likely to be younger and current drinkers, to walk 1 h or more per week, to have higher education, and histories of diabetes and hypertension, and less likely to be current smokers. Over the 741,706 person-years of follow-up (median follow-up period was 19.1 years) for 45,837 male subjects, we found that a total of 268 participants died from COPD.

**Table 1:** Participants’ age-adjusted baseline demographics according to body mass index.

Body mass index* (kg/m^2^)	< 18.5	18.5 to < 20.0	20.0 to < 22.0	≥ 22.0	*p* for difference
No. at risk	2404	5069	12,127	26,237	
Age, mean (SD)	61.4 (14.3)	55.9 (13.5)	54.8 (12.7)	53.4 (11.7)	< .0001
Body mass index (kg/m^2^)	17.5	19.3	21.0	24.5	< .0001
Alcohol intake (g/day)	30.8	33.6	33.6	34.4	< .0001
Smoking index (pack x year)	33.9	32.6	32.5	33.0	< .0001
Never smoker (%)	16.6	16.8	18.7	22.4	< .0001
Current smoker (%)	61.9	63.4	59.4	51.8	< .0001
Walking, 1 h/week or over (%)	51.8	47.8	47.9	52.8	< .0001
Exercise, 5 h/week or over (%)	7.4	7.9	6.9	6.4	0.002
High mental stress (%)	24.4	23.9	23.6	25.1	0.053
College education or higher (%)	15.5	16.5	17.8	19.6	< .0001
History of diabetes mellitus (%)	6.5	5.3	5.2	6.1	< .0001
History of hypertension (%)	16.0	14.2	15.1	20.1	< .0001

Table 2 represents age-adjusted and multivariate-adjusted HRs (95% CI) of mortality from COPD according to BMI categories. The multivariate-adjusted excess risk of COPD death was 3.2 times higher among subjects in the lowest BMI category (BMI < 18.5 kg/m^2^), compared to those with a BMI between 20.0 and < 22.0 kg/m^2^. There was a 42% reduction in risk for subjects in the highest BMI category (BMI ≥ 22.0 kg/m^2^). Further, the multivariate HR and 95% CI for COPD mortality associated with a 1-SD decrement of BMI (2.80 kg/m^2^) was 0.48 (0.41 to 0.57). The inverse associations were not attenuated after adjusting for potential confounding variables.

**Table 2:** Hazard ratios (HRs) and 95% confidence intervals of COPD mortality according to body mass index.

Body mass index (kg /m^2^)	< 18.5	18.5 to < 20.0	20.0 to < 22.0	≥ 22.0	/ 1-SD body mass index (2.80 kg/m^2^)
No. at risk	2,404	5,069	12,127	26,237	
Person-years	31,413	78,378	195,524	436,391	
No. of COPD deaths	73	63	73	59	
Age-adjusted HRs	4.33 (3.11–6.01)	2.07 (1.48–2.90)	1.00	0.43 (0.31–0.61)	0.38 (0.33–0.44)
Multivariate HRs	3.24 (2.31–4.54)	1.68 (1.20–2.37)	1.00	0.58 (0.41–0.83)	0.48 (0.41–0.57)
Analysis excluding deaths from COPD during the first 5 years					
No. at risk	2,051	4,739	11,518	25,298	
Person-years	30,507	77,460	193,784	433,735	
No. of COPD deaths	56	56	71	56	
Age-adjusted HRs	3.67 (2.58–5.23)	1.91 (1.35–2.72)	1.00	0.42 (0.29–0.59)	0.42 (0.36–0.49)
Multivariate HRs	2.81 (1.96–4.05)	1.57 (1.10–2.24)	1.00	0.57 (0.39–0.81)	0.53 (0.45–0.62)
Analysis excluding deaths from COPD during the first 10 years					
No. at risk	1,696	4,296	10,636	23,685	
Person-years	27,866	74,136	187,156	421,441	
No. of COPD deaths	35	43	55	46	
Age-adjusted HRs	3.35 (2.19–5.13)	1.94 (1.30–2.89)	1.00	0.43 (0.29–0.64)	0.45 (0.38–0.54)
Multivariate HRs	2.61 (1.68–4.04)	1.60 (1.07–2.39)	1.00	0.61 (0.41–0.92)	0. 58 (0.48–0.70)
Analysis excluding deaths from COPD during the first 15 years					
No. at risk	1,413	3,848	9,717	22,023	
Person-years	24,386	68,598	175,641	400,627	
No. of COPD deaths	17	25	37	28	
Age-adjusted HRs	2.74 (1.54–4.86)	1.73 (1.04–2.87)	1.00 0.38 (0.23–0.62)		0.46 (0.37–0.58)
Multivariate HRs	2.46 (1.36–4.45)	1.51 (0.90–2.52)	1.000.53 (0.32–0.88)		0.57 (0.45–0.74)

Multivariate adjustment for age, weight change from the age of 20, ethanol intake, hours of walking, hours of exercise, education history, smoking index, and disease histories.

**Figure 1:**
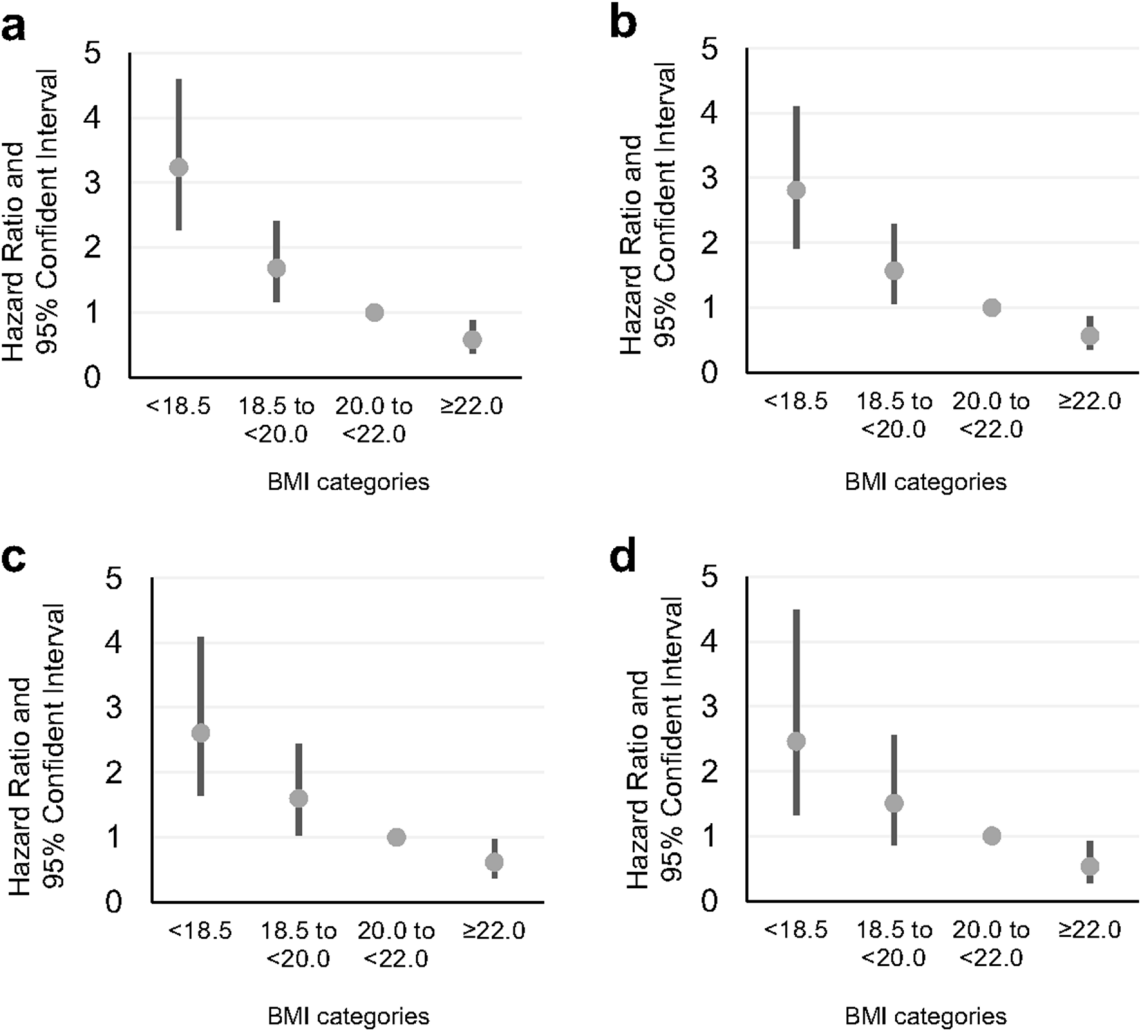
Hazard ratios (HR) and 95% confidence intervals (95% CI) of COPD mortality according to the four BMI categories. HR (95% CI) of the total population (**a**), and after excluding events within 5 years (**b**), 10 years (**c**) and 15 years (**d**), are shown.

In addition, when COPD deaths within 5, 10, and 15 years of the baseline survey were excluded, the inverse association remained statistically significant (Table 2) and the HR trends were similar (Fig. 1). This inverse association was also detected regardless of expectoration reported in the baseline survey (Supplementary Table S1 online). Of 268 deaths, 6.3% were of never smokers. When COPD deaths among smokers were analysed the inverse association between lower BMI and greater HR remained significant (Supplementary Table S2 online).

Table 3 shows participants’ demographic characteristics according to changes in weight from age 20. Compared to the control group whose weight change was > − 5.0, < 5.0 kg, those who lost ≥ 10.0 kg were more likely to be older, current smokers, to do exercise more, and to have lower mental stress and histories of diabetes and hypertension, and less likely to have higher education. On the other hand, those who gain ≥ 5.0 kg were more likely to be younger, never smokers, to walk 1 h or more per week, to have high mental stress, higher education, and history of hypertension.

**Table 3:** Participants’ age-adjusted baseline demographics according to weight change from the age of 20.

Weight change (kg) *	≤ − 10.0	-10.0 < , ≤ − 5.0	− 5.0 < , < 5.0	≥ 5.0	*p* for difference
Weight loss (kg)	Weight loss ≥ 10.0	5.0 ≤ , weight loss < 10.0	− 5.0 < weight loss < 5.0	Weight gain ≥ 5.0 weight gain ≥ 5.0	
No. at risk	2296	4504	12,682	11,941	
Age	66.2 (10.2)	61.8 (10.4)	54.0 (11.8)	51.1 (10.8)	< .0001
Body mass index (kg/m^2^)	20.3	21.1	21.8	24.7	< .0001
Alcohol intake (g/day)	35.0	34.2	33.7	33.4	0.07
Smoking index (pack × year)	39.2	36.4	31.7	31.6	< .0001
Never smoker (%)	14.1	16.3	21.1	22.8	< .0001
Current smoker (%)	63.1	60.8	57.6	49.9	< .0001
Walking, 1 h/week or over (%)	46.9	45.1	48.2	57.8	< .0001
Exercise, 5 h/week or over (%)	10.8	9.9	6.5	5.4	< .0001
High mental stress (%)	16.4	18.5	24.2	29.9	< .0001
College education or higher (%)	13.6	14.3	18.5	23.8	< .0001
History of diabetes mellitus (%)	9.5	7.5	5.0	5.5	0.29
History of hypertension (%)	24.1	21.3	15.6	18.9	< .0001

Table 4 shows the HR for COPD death according to weight changes from the age of 20. The multivariate-adjusted HR in the first weight change category (participants who had lost 10 kg or more) was 3.3 times greater than in the reference category (participants who had lost or gained less than 5 kg). The age-adjusted HR in the fourth category (participants who had gained 5 kg or more) was 47% lower than that of the reference category (Table 4). After adjusting for confounding variables, the multivariate-adjusted HR and 95% CI of COPD mortality associated with a 1-SD decrement of weight change was 0.63 (0.59–0.68).

**Table 4:** Hazard ratios (HRs) and 95% confidence intervals of COPD mortality according to weight change from the age of 20.

Weight change (kg)	≤ − 10.0	− 10.0 < , ≤ − 5.0	− 5.0 < , < 5.0	≥ 5.0	/1-SD of weight change (8.49 kg)
Weight loss (kg)	Weight loss ≥ 10.0	5.0 ≤ , weight loss < 10.0	− 5.0 < weight loss < 5.0	Weight gain ≥ 5.0	
No. at risk	2296	4504	12,682	11,941	
Person-years	28,511	65,912	207,334	201,702	
No. of COPD deaths	81	49	48	11	
Age-adjusted HRs	5.99 (4.13–8.69)	1.93 (1.29–2.88)	1.00	0.31 (0.16–0.61)	0.66 (0.62–0.70)
Multivariate HRs	3.34 (2.25–4.96)	1.38 (0.91–2.08)	1.00	0.53 (0.26–1.05)	0.63 (0.59–0.68)
Analysis excluding deaths from COPD during the first 5 years					
No. at risk	1937	4163	12,179	11,642	
Person-years	27,567	64,964	205,878	200,824	
No. of COPD deaths	65	45	46	11	
Age-adjusted HRs	5.34 (3.61–7.91)	1.88 (1.24–2.86)	1.00	0.33 (0.17–0.63)	0.67 (0.63–0.72)
Multivariate HRs	2.94 (1.94–4.46)	1.32 (0.86–2.02)	1.00	0.57 (0.29–1.15)	0.68 (0.60–0.78)
Analysis excluding deaths from COPD during the first 10 years					
No. at risk	1526	3618	11,412	11,130	
Person-years	24,451	60,876	200,024	196,949	
No. of COPD deaths	42	35	37	7	
Age-adjusted HRs	4.96 (3.14–7.84)	1.93 (1.21–3.08)	1.00	0.25 (0.11–0.57)	0.68 (0.63–0.73)
Multivariate HRs	2.82 (1.73–4.59)	1.38 (0.86–2.23)	1.00	0.45 (0.19–1.04)	0.67 (0.58–0.76)
Analysis excluding deaths from COPD during the first 15 years					
No. at risk	1174	3076	10,630	10,597	
Person-years	20,099	54,102	190,218	190,248	
No. of COPD deaths	19	17	28	5	
Age-adjusted HRs	3.30 (1.82–6.01)	1.30 (0.71–2.39)	1.00	0.24 (0.09–0.61)	0.71 (0.63–0.79)
Multivariate HRs	1.90(1.01–3.60)	0.93 (0.50–1.73)	1.00	0.37 (0.14–1.03)	0.74 (0.59–0.92)

Multivariate adjustment for age, BMI, ethanol intake, hours of walking, hours of exercise, education history, smoking index, and disease histories.

There were significant inverse trends in COPD mortality associated with weight changes. These inverse associations remained unchanged after the exclusion of participants who died within 5, 10 and 15 years of the baseline survey (Table 4), after stratified by initial expectoration symptoms (Supplementary Table S3 online), ever-smokers (Supplementary Table S4 online), and smoking index < 20 or ≥ 20 (Supplementary Table S5 online). Likewise, after stratification with the four categories of BMI levels, multivariate-adjusted HRs and 95% CI of COPD mortality associated with a 1-SD increment of weight change were mostly similar among the four BMI categories, ranging 0.65 (0.51–0.84) in those with BMI < 18.5 (mean(SD) = 17.5(0.9)kg/m^2^) to 0.75 (0.61–0.91) in those with BMI ≥ 22.0 (mean(SD) = 14.5(2.1)kg/m^2^), and the association remains statistically significant exclusively in weight loss of ≥ 10.0 kg, and not of ≥ 5 to < 10.0 kg (Supplementary Table S6 online).

**Figure 2:**
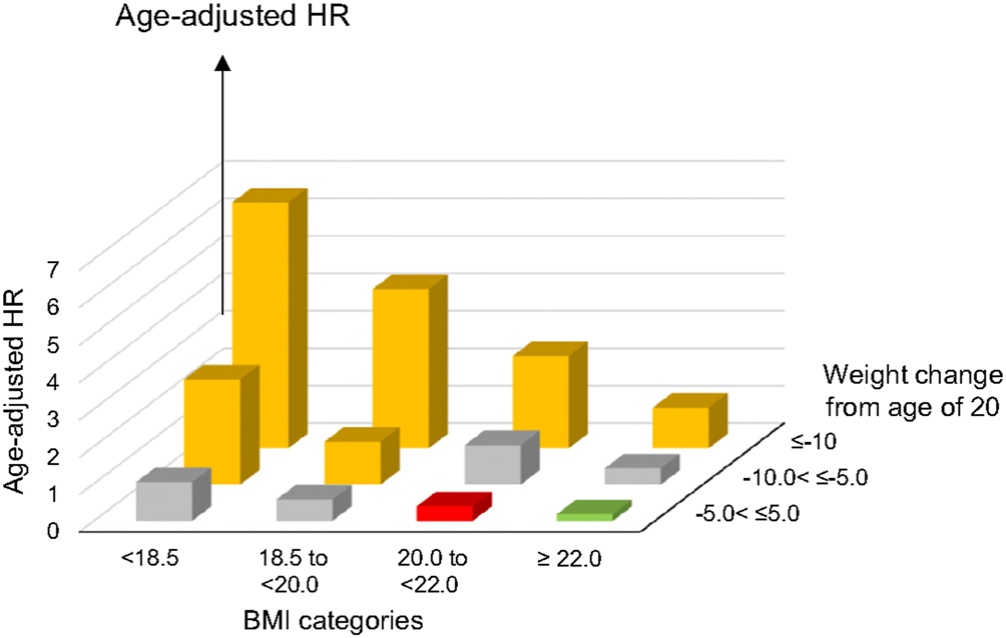
Hazard ratio (HR) of COPD mortality based on the four BMI and four weight change categories. The red bar represents the reference group (5.0 < , < 5.0 kg weight change and 20.0 to < 22.0 kg/m^2^ BMI). Yellow bars indicate categories with significantly greater HR, grey bars represent categories that did not show statistically significant difference, and green bars represent a statistically significant smaller HR compared to the reference group. This figure suggests that BMI and weight loss are independent risks for COPD death.

Figure 2 presents the association of COPD mortality—represented by age-adjusted HRs—with both BMI and weight change categories, referenced to those with BMI between 20.0 and < 22.0 kg/m^2^ and weight change between >  − 5.0 and < 5.0 kg. The greatest risk of COPD mortality was found among participants in the lowest BMI and greatest weight loss groups (BMI < 18.5 kg/m^2^ and weight loss ≥ 10 kg). The HR (95% CI) for these participants was 11.2 (6.33–19.7). Participants in the highest BMI and largest weight gain groups (BMI ≥ 22.0 and weight change ≥ 5) had the lowest risk for COPD mortality. The HR (95% C.I.) for this category of participant was 0.28 (0.12–0.63). These results shown in Fig. 2 suggest that weight change from age of 20 and BMI values have an additive and inverse effect on COPD mortality. Of note, even the participants with BMI > 22.0 kg/m^2^ and weight loss ≥ 10.0 kg had significantly greater HR compared to the reference.

## Discussion

In the present large-scale prospective community-based cohort study, lower BMI values and greater weight loss were independently associated with a greater risk of COPD mortality. The associations were observed even after exclusion of 5-, 10-, and 15-year early mortality incidents and participants with expectoration symptoms. Furthermore, by analysing COPD mortalities together with a non-COPD population (after excluding those with a history of tuberculosis), our study convincingly determined that low BMI and weight loss are associated with the increased risk of COPD mortality.

The previous Chinese study implied that lower BMI was associated with COPD mortality partially due to the comorbid chronic tuberculous infection^11^. However, our present analyses found an inverse association between BMI and COPD mortality regardless of a history of tuberculosis, suggesting that tuberculosis per se may not be responsible for the association. Nonetheless, the association between lower BMI and higher mortality from COPD even after exclusion of first 5, 10 and 15 years in the present study may suggest that being underweight may have a direct effect on the manifestation of COPD mortality. Furthermore, the inverse associations between BMI and COPD mortality did not differ by smoking status in the present study, although smoking may lower body weight^14^ and increase risk of COPD^15^. This suggests that smoking is unlikely to be an intermediate factor for the presented association.

There could be several possible explanations for the risk elevation of COPD mortality among underweight men. Firstly, underweight men may be less resistant to infections, such as pneumonia^16^, and pneumonia is known to initiate fatal exacerbations of COPD. In addition, underweight COPD patients are more likely to suffer from other comorbidities, such as cardiovascular diseases, osteoporosis, depression, and lung cancer^15,17,18^, in association with a worse prognosis^19^. Secondly, underweight patients with anorexia nervosa showed emphysematous changes , a typical finding of COPD, in CT images^20^, as well as an old description of the residents with restricted intake of protein and calorie by physicians in the Warsaw ghetto^21^, suggesting that being underweight or having poor nutrition may develop COPD. Finally, the association between low BMI and COPD mortality may be explained by the accelerating effect that chronic cigarette smoke exposure has on the lung ageing process^22–24^. Both COPD patients (mostly smokers) and the elderly have been shown to suffer from low body weight due to reduced free fat mass^5,25–28^. A reduction in Sirtuin-1 activity and cellular senescence has also been associated with the development of COPD and cigarette smoke exposure^22–24^. Thus, chronic smoke exposure artificially induces ageing processes that result in both low body weight and lung ageing, thus potentially leading to the strong association between having low BMI and higher COPD mortalities, as observed in the present study.

A recent analysis of the ECLIPSE cohort comprising COPD patients with GOLD stages 2 to 4 showed that COPD mortality was predicted with the presence of cachexia, which is defined as rapid weight loss > 5% or > 2% if BMI < 20 kg/m^2^ within 12 months^29^. This study raises two issues that require discussion. Firstly, the association between weight reduction and greater COPD mortality in our study may be accounted for by cachexia. Unfortunately, it was not assessed whether or not the present study included the participants with cachexia, who developed weight loss rapidly within a year^30^, however weight reduction could occur in the early stages of COPD, since the association remained statistically significant, even after excluding COPD death in the initial 5, 10 and 15 years of our study (Table 4), suggesting that weight reduction is risk for COPD mortality.

Secondly, the definition of cachexia in the ECLIPSE study^29^ suggests that a weight reduction > 2% in the patients with lower BMI has the same impact on mortality as that > 5% in those with normal BMI. However, our study showed that HRs with 1-SD decrement of weight change were not so different between the four BMI categories (Supplementary Table S6 online), and therefore the impact of percentage of weight change to BMI on COPD mortality is independent from the four BMI categories. This implies that the weight reduction since the age of 20 in the present study may not due to cachexia.

### Political implication

There are remarkable disparities between Asian nations in the proportion of different types of respiratory diseases and mortality, including COPD, tuberculosis, asthma and pneumonia for total respiratory mortality^11–13^. A large cohort study of 220,000 Chinese men aged 40 to 79 year with 15 years follow-up showed that 5 kg/m^2^ decrement of BMI was associated with 31% higher risk of mortality from COPD^11^. This Chinese cohort study had the smaller excess risk of COPD mortality than did our present study, which estimated 60% increase in COPD mortality per 2.8 kg/m^2^ decrement of BMI, equivalent to 107% increase per 5.0 kg/m^2^ decrement.

A study described the harmful effects of cigarette smoking, and found that it was the largest cause of non-communicable disease (NCD) mortality—deaths caused by cardiovascular and respiratory diseases, and cancer^31^. Cigarette smoking accounted for 15% of total NCD deaths in Japanese men, followed by high blood pressure^31^. Confirming this, our study found that the majority of COPD mortalities occurred in smokers. Weight reduction and smoking cessation have long been the mainstays in efforts to prevent NCD. The present study did not collect information regarding whether the weight loss resulted from voluntary activity or involuntarily from disease processes. There are several voluntary means of weight reduction, including diet^32^, exercise^33^ and behavioural modification^34^, whereas involuntary processes in COPD patients include imbalance in energy, impaired oxygenation and appetite loss-linked reduced dietary intake^35,36^, as well as cachexia^37^ and muscle wasting (sarcopenia)^38^. These different causes of weight change in COPD patients can indirectly affect the association between the weight change and mortality from COPD. Nevertheless, the present study may provoke a possible dilemma as voluntary weight loss may reduce mortality due to cardiovascular diseases, but increase COPD mortality (i.e. the obesity paradox)^39^.

### Limitations

There are several limitations in this study; first, weight change was defined as the difference between body weight at the baseline survey and that at the age of 20. Because the latter value depends on participants’ memory, the possible bias was present in reported values. Specifically, thin men may have overestimated their past weight, while overweight men may have underestimated it^40^. However, the recalled and measured data were well correlated^40,41^. Moreover, our previous study^16^ had confirmed the results of other studies in terms of the association between weight loss and mortality from all causes^12,42,43^. Second, our study only included men, which might restrict the generalizability of the results to women or individuals of other ethnicities. On the other hand, the prevalence of asthma and tuberculosis in Japan is not as high as in other Asian countries. Thus, our study could provide comparative evidence to that carried out in Western countries, where tuberculosis is not a leading cause of death. Third, there is a potential for reverse causation between having low BMI, weight loss, and higher COPD mortality risk. However, the possible reverse causation may be small in the present study, since these inverse associations remained unchanged after the exclusion of mortality events occurring within the first 5, 10 and 15 years. Finally, we did not make any lung function assessment of the participants, so we are not able to discuss the severity of COPD. However, analyses with stratification by smoking exposure (≥ 20 pack year vs < 20) at enrollment showed that the inverse association between BMI and mortality from COPD was observed independently from smoking. This indirectly suggested that lower BMI may predict worse mortality regardless of any reduction in lung function, since COPD patients with more impaired lung fucntion are more likely to belong to the group with a higher smoking index .

Our study presents evidence of an inverse and additive association between both weight change from the age of 20 and BMI, and COPD mortality (Fig. 2). Low BMI and weight loss may be noticeable long before COPD is diagnosed, or COPD-linked symptoms are observed. We may thus be able to advocate for low body weight and weight loss as possible predictive markers of COPD mortality, even if they might not be a pathogenic cause of disease development.

### Strengths of the study

A major strength of our study was that the JACC study followed a large prospective well-characterized cohort of the general population for a long period^16,44–47^. This enabled us to analyse the data using the combined four BMI, and four weight change categories after adjustment with many variables, as well as to conduct further stratified analyses by smoking status and initial symptoms.

## Conclusion

In conclusion, our large-scale prospective community-based epidemiological study showed that lower BMI and weight loss are potential independent disease-specific markers for COPD mortality. The individuals who have lost more than 10 kg since the age of 20, and those with a BMI < 18.5 kg/m^2^ had highest risk for mortality from COPD.

## Methods

### Study population and mortality surveillance

The study population was drawn from participants in the Japan Collaborative Cohort Study for Evaluation of Cancer Risk (JACC) study^44^, which was sponsored by Monbu-kagaku-sho (the Ministry of Education, Science, Sports and Culture, Japan). This study’s baseline survey and participant follow-up methodology has been described in detail elsewhere^44^. Briefly, a comparative study was conducted from 1988 to 1990 with 110,585 individuals (46,395 men and 64,190 women) aged 40 to 79 years who were living in 45 communities throughout Japan. Participants completed self-administered questionnaires that asked about their current weight and height, their weight at the age of twenty, their lifestyles, and any medical history of cardiovascular disease (CVD) and cancer^44^ and tuberculosis. Informed consent was obtained from participants before they completed the questionnaire or sometimes from community leaders instead of individuals, as this had been a common practice for informed consent at that time in Japan.

Since mortality due to COPD was rather rare among the female participants, we focused on male JACC study participants. We excluded 8094 male participants who had a history of lethal diseases including stroke, myocardial infarction, and cancer, as well as tuberculosis, leaving a total of 45,837 male subjects enrolled in the study. The analyses were conducted at 2019.

Of 45 municipalities, participants were followed until the end of 2009 to determine COPD-caused mortality in 35 areas. Of remaining 10 areas, four areas terminated follow-up in 1999, four in 2003, and two in 2008. Mortality data were centralized at the Ministry of Health and Welfare, and the underlying cause of death was coded according to the International Classification of Diseases (ICD), 9th version, for deaths between 1988 and 1994, and the 10th revision for deaths from 1995 and thereafter.

Registration of death is required by the Family Registration Law of Japan, and thus all deaths in the cohort were ascertained with death certificates, under the permission of the Director General of the Prime Minister’s Office (Ministry of Internal Affairs and Communications). The only exceptions were subjects who died after they moved away from their original community. These were treated as censored cases. In this study, COPD mortality is defined as that from emphysema (ICD10 J43) and from other diseases or conditions in association with COPD (ICD10 J44). The relevant outcome was defined as death attributed to COPD during the study period. For each participant, the follow up period (person-year) was the period between completing the baseline survey and death, emigration, or the end of the study, i.e., the end of 1999, 2003, 2008, or 2009.

The study population was drawn from participants in the Japan Collaborative Cohort Study for Evaluation of Cancer Risk (JACC) study^48^ and COPD mortality is defined as that from emphysema (ICD10 J43) and from other diseases or conditions in association with COPD (ICD10 J44) in death certificates.

### Statistical analysis

Participants were divided into four categories according to their BMI (kg/m^2^): (1) BMI less than 18.5, (2) BMI from 18.5 to < 20.0, (3) BMI from 20.0 to < 22.0, and (4) BMI of 22.0 or above, based on a previously published study^47^, so that tthese groupings presented a similar number of COPD deaths across BMI categories (Table 1). Category 3 (20.0 to < 22.0) was treated as the reference group. The analyses of weight change from the age of 20 were based on our previous studies^45,49^. Briefly, weight changes were defined as the difference between body weight at the baseline survey and that at the age of 20, and the participants were divided into four groups as in our previous studies^45,49^: (1) participants who had lost 10 kg or more (≤ − 10), (2) those who had lost between 5 to < 10 kg (> − 10, ≤  − 5), (3) those who had either lost or gained less than 5 kg (> − 5, <  + 5) (reference), and (4) those who had gained 5 kg or more (≥ + 5 ).

Means with standard deviations (SD) and proportions of selected COPD risk factors were determined, according to categories of BMI, as well as to weight change. To test the difference between these categories, t-test and chi square test were performed. Age-adjusted and multivariate adjusted hazard ratios (HRs) and their 95% confidence intervals (95% CI), as well as 1—standard deviation (SD) increments of BMI or weight change, were determined using Cox proportional hazard models. Then, participants were further divided into 16 groups based on the combination of BMI and weight change categories, and each HR (95% CI) was determined and referenced to the BMI (20.0 to < 22.0) and weight change (> − 5, <  + 5) groups. The crude death ratio (/100,000) and age-adjusted HR using the Cox proportional hazard model were also determined.

Potential variables in the multivariate adjusted model included age, BMI, weight change from age of 20, ethanol intake, hours of walking per week, hours of exercise per week, education level, perceived mental stress, history of hypertension, and history of diabetes (all values as recorded in the baseline survey). We conducted the stratified analyses by smoking status and the presence or absence of initial symptoms of expectoration, as well as by BMI categories (< 18.5, ≥ 18.5 to < 20.0, ≥ 20.0 to < 22.0, ≥ 22.0 kg/m^2^) at base line survey. Early deaths caused by COPD—those that took place within the first 5, 10, and 15 years of enrolment—and participants with self-reported expectoration at the baseline survey were excluded in order to reduce the chances of a reverse causal relationship between BMI or weight reduction, and COPD mortality.

All analyses were performed using SAS version 9.3 (SAS Institute, Cary, North Carolina, USA). Two-tailed probability p-values lower than 0.05 were considered statistically significant.

All the study was carried out in accordance with the ethics guideline set out by the Ministry of Health, Labour and Welfare, Japan, based on the fundamental principle in the Declaration of Helsinki^50^, and all of these processes were reviewed and approved by the Ethics Committees of the Hokkaido University, Osaka University and Juntendo University (Ethical Review Board of Juntendo University Faculty of Medicine). The data will be available on request.

## Supplementary information

Supplementary Information.

## Abbreviations

COPD: Chronic obstructive pulmonary disease
BMI: Body mass index
HR: Hazard ratio
