# Supplementary material for: Low BMI and weight loss aggravate COPD mortality in men, findings from a large prospective cohort: the JACC study
Source: Sci Rep. 2021 Jan 15;11:1531. doi: 10.1038/s41598-020-79860-4 (PMC7810869; doi:10.1038/s41598-020-79860-4)
Supplement: Supplementary file 1 — Supplementary Information. [file 41598_2020_79860_MOESM1_ESM.pdf]

Low BMI and weight loss aggravate COPD mortality in men, findings from a large prospective cohort, The JACC Study

Hiroo Wada[1], Ai Ikeda[1], Koutatsu Maruyama[2], Kazumasa Yamagishi[3], Peter J Barnes[4], Takeshi Tanigawa[1], Akiko Tamakoshi[5], Hiroyasu Iso[6, *]

[1]Department of Public Health, Juntendo University Graduate School of Medicine, Bunkyo, Tokyo, Japan
[2] Department of Bioscience, GraduateSchool of Agriculture, Ehime University, Matsuyama, Ehime Japan
[3]Department of Public Health Medicine, Faculty of Medicine, and Health Services Research and Development Center, University of Tsukuba, Tsukuba, Ibaraki, Japan
[4]National Heart & Lung Institute, Imperial College London, London, UK
[5]Department of Public Health, Hokkaido University
[6]Department of Social and Environmental Medicine, Graduate School of Medicine, Osaka University, Suita, Osaka, Japan

*Corresponding author: Hiroyasu Iso, Professor and Chair
Public Health, Department of Social and Environmental Medicine, Osaka University Graduate School of Medicine,
2-2 Yamada-oka, Suita, Osaka 565-0871,
Japan
Phone 06-6879-3911
Electronic address: iso@pbhel.med.osaka-u.ac.jp

Low BMI and weight loss aggravate COPD mortality in men,

findings from a large prospective cohort, The JACC Study

Hiroo Wada[1], Ai Ikeda[1], Koutatsu Maruyama[1], Kazumasa Yamagishi[2],

Peter J Barnes[3], Takeshi Tanigawa[1], Akiko Tamakoshi[4], Hiroyasu Iso[5, *]

[1]Department of Public Health, Juntendo University Graduate School of Medicine,

Bunkyo, Tokyo, Japan

[2]Department of Public Health Medicine, Faculty of Medicine, University of Tsukuba,

Tsukuba, Ibaraki, Japan
[3]National Heart & Lung Institute, Imperial College London, London, UK
[4]Department of Public Health, Hokkaido University
[5]Department of Social and Environmental Medicine,
Graduate School of Medicine, Osaka University, Suita, Osaka, Japan

Table S1. Hazard ratios (HRs) and 95% confidence intervals of COPD mortality according to body mass index, stratified by expectoration

Body mass index (kg /m^2)	<18.5			18.5 to <20.0			20.0 to <22.0	≥22.0			/ 1-SD body mass index (2.80 kg/m^2)		
Sputum (-)													
No. at risk	843			1,873			4,571	9,708					
Person-years	11,886			29,870			75,346	163,938					
No. of COPD deaths	18			20			15	13					
Age-adjusted HRs	5.95	(2.98	-11.89)	3.02	(1.55	-5.91)	1.00	0.48	(0.23	-1.02)	0.33	(0.24	-0.44)
Multivariate HRs	5.10	(2.50	-10.40)	2.76	(1.39	-5.47)	1.00	0.61	(0.28	-1.32)	0.36	(0.26	-0.51)
Sputum (+)													
No. at risk	1,130			2,219			5,299	11,603					
Person-years	13,707			33,134			83,215	189,472					
No. of COPD deaths	45			37			48	36					
Age-adjusted HRs	3.73	(2.47	-5.62)	1.94	(1.26	-2.98)	1.00	0.41	(0.26	-0.63)	0.40	(0.34	-0.48)
Multivariate HRs	2.63	(1.71	-4.05)	1.54	(1.00	-2.38)	1.00	0.58	(0.37	-0.90)	0.53	(0.44	-0.65)

Multivariate adjustment for age, weight change from age of 20, ethanol intake, hours of walking, hours of exercise, education history, smoking index, and disease histories.

Table S2. Hazard ratios (HRs) and 95% confidence intervals of COPD mortality according to body mass index in ever-smokers

Body mass index (Kg/m^2)	<18.5			18.5 to <20.0			20.0 to <22.0	≥22.0			/ 1-SD body mass index (2.80kg/m^2)		
Ever smoker													
No at risk	1,887			4,006			9,422	19,373					
Person-years	24,555			61,732			151,443	320,950					
No. of COPD deaths	58			56			66	53					
Age-adjusted HRs	3.88	(2.72	-5.55)	2.06	(1.44	-2.94)	1.00	0.47	(0.33	-0.68)	0.42	(0.36	-0.49)
Multivariable HRs	3.76	(2.63	-5.40)	1.97	(1.37	-2.82)	1.00	0.49	(0.34	-0.70)	0.43	(0.37	-0.50)

Multivariate adjustment for age, ethanol intake, hours of walking, hours of exercise, education history, smoking index and disease histories.

Table S3. Hazard ratios (HRs) and 95% confidence intervals of COPD mortality according to weight change in patients with expectoration

Weight change (kg)	≤ -10.0			-10.0<, ≤ -5.0			-5.0<, <5.0	≥ 5.0			/1-SD of weight change (8.49 kg)		
weight loss (kg)	weight loss ≥ 10.0			5.0 ≤, weight loss <10.0			-5.0 < weight loss <5.0	weight gain ≥ 5.0					
No. at risk	1,291			2,400			6,643	6,492					
Person-years	15,532			34,459			106,432	108,101					
No. of COPD deaths	60			36			34	7					
Age-adjusted HRs	5.73	(3.69	-8.90)	1.97	(1.23	-3.17)	1.00	0.27	(0.12	-0.60)	0.61	(0.57	-0.66)
Multivariate HRs	3.31	(2.06	-5.32)	1.49	(0.92	-2.42)	1.00	-	-		0.62	(0.54	-0.72)

Multivariate adjustment for age, BMI, ethanol intake, hours of walking, hours of exercise, education history, smoking index, and disease histories.

Table S4. Hazard ratios (HRs) and 95% confidence intervals of COPD mortality according to weight change in ever-smokers

Weight change (kg)	≤ -10.0			-10.0<, ≤ -5.0			-5.0<, <5.0	≥ 5.0			/1-SD of weight change (8.49 kg)		
weight loss (kg)	weight loss ≥ 10.0			5.0 ≤, weight loss <10.0			-5.0 < weight loss <5.0	weight gain ≥ 5.0					
No at risk	1,825			3,548			9,566	8,877					
Person-years	22,830			51,952			155,328	149,152					
No. of COPD deaths	70			40			42	11					
Age-adjusted HRs	5.48	(3.69	-8.16)	1.69	(1.09	-2.62)	1.00	0.36	(0.19	-0.70)	0.67	(0.62	-0.72)
Multivariate HRs	3.21	(2.10	-4.90)	1.23	(0.78	-1.92)	1.00	0.59	(0.29	-1.19)	0.67	(0.59	-0.76)

Multivariate adjustment for age, BMI, ethanol intake, hours of walking, hours of exercise, education history, smoking index, and disease histories.

Table S5. Hazard ratios (HRs) and 95% confidence intervals of COPD mortality according to body mass index, after stratified by heavy smokers (SI≥ 20 pack years) or not (SI< 20 pack years)

Body mass index (kg /m^2)	<18.5			18.5 to <20.0			20.0 to <22.0	≥22.0			/ 1-SD body mass index (2.80 kg/m^2)		
Smoking Index <20													
No. at risk	1,366			2,858			7,118	16,667					
Person-years	17,984			44,451			115,963	278,783					
No. of COPD deaths	40			27			29	22					
Age-adjusted HRs	5.39	(3.32	-8.75)	2.31	(1.37	-3.91)		0.37	(0.21	-0.65)	0.34	(0.27	-0.42)
Multivariate HRs	3.87	(2.35	-6.37)	1.82	(1.07	-3.10)		0.51	(0.29	-0.90)	0.43	(0.34	-0.54)
Smoking Index ≥20													
No. at risk	1,038			2,211			5,009	9,570					
Person-years	13,429			33,927			79,561	157,609					
No. of COPD deaths	33			36			44	37					
Age-adjusted HRs	3.56	(2.26	-5.62)	1.82	(1.17	-2.83)		0.55	(0.36	-0.86)	0.46	(0.38	-0.56)
Multivariate HRs	2.84	(1.77	-4.55)	1.60	(1.02	-2.49)		0.65	(0.41	-1.03)	0.53	(0.42	-0.66)

Multivariate adjustment for age, weight change from age of 20, ethanol intake, hours of walking, hours of exercise, education history, smoking index, and disease histories.

Table S6. Hazard ratios and 95% confidence interval of COPD mortality according to weight change from age of 20, stratified by BMI categories

BMI	≤ -10.0			-10 to ≤5			>-5 to <5.0	≥5			/1-SD WC		
<18.5													
No at risk	533			496			569	18					
Person year	5,380			6,371			8,889	219					
COPD death	35			14			6	0					
age-adjusted	3.99	(1.61	- 9.92)	1.78	(0.67	- 4.70)	ref	-	-	-	0.48	(0.35	- 0.66)
multi-adjusted	4.12	(1.65	- 10.29)	2.17	(0.81	- 5.82)	ref	-	-	-	0.57	(0.41	- 0.79)
18.5 to <20.0													
No at risk	565			954			1,699	141					
Person year	6,960			13,855			27,809	2,360					
COPD death	24			11			10	0					
age-adjusted	3.74	(1.72	- 8.12)	1.14	(0.48	- 2.71)	ref	-	-	-	0.51	(0.35	- 0.76)
multi-adjusted	3.47	(1.56	- 7.75)	0.95	(0.39	- 2.32)	ref	-	-	-	0.52	(0.34	- 0.80)
20.0 to <22.0													
No at risk	647			1,691			4,687	1,238					
Person year	8,398			25,150			77,401	20,712					
COPD death	16			18			20	2					
age-adjusted	3.44	(1.73	- 6.83)	1.54	(0.81	- 2.96)	ref	0.46	(0.11	- 1.96)	0.52	(0.37	- 0.75)
multi-adjusted	2.70	(1.33	- 5.46)	1.43	(0.74	- 2.75)	ref	0.39	(0.09	- 1.76)	0.57	(0.39	- 0.82)
≥22.0													
No at risk	551			1,363			5,727	10,544					
Person year	7,774			20,536			93,235	178,412					
COPD death	6			6			12	9					
age-adjusted	3.86	(1.43	- 10.41)	1.50	(0.56	- 4.02)	ref	0.61	(0.25	- 1.45)	0.74	(0.62	- 0.89)
multi-adjusted	3.27	(1.18	- 9.00)	1.33	(0.49	- 3.60)	ref	0.58	(0.23	- 1.45)	0.74	(0.60	- 0.90)
